# Impact of heatwaves on depression and anxiety in India: A time-series analysis

**DOI:** 10.6026/973206300214301

**Published:** 2025-12-15

**Authors:** Murali M.R, Rahul Tiwari, Gayathri Priyadarshini R.S, Heena Dixit Tiwari, Prashant M.C, Tohid Ali, Afroz Kalmee Syed

**Affiliations:** 1 Department of Psychiatry, JSS Academy of Higher Education & Research, Mysore, Karnataka, India; 2 Department of Oral and Maxillofacial Surgery, RKDF Dental College and Research Centre, Sarvepalli Radhakrishnan University, Bhopal, Madhya Pradesh, India; 3 Department of Pathology, Sree Balaji Medical College and Hospital, BIHER University, Chrompet, Chennai, Tamil Nadu, India; 4 Programme Officer, Blood Cell, Commissionerate of Health and Family Welfare, Government of Telangana, Hyderabad, India; 5 Department of Oral Medicine and Radiology, RKDF Dental College and Research Centre, Sarvepalli Radhakrishnan University, Bhopal, Madhya Pradesh, India; 6 Department of Oral and Maxillofacial Pathology, Writing and Publications, Tenali, Andhra Pradesh, India

**Keywords:** Heatwaves, depression, anxiety, hospitalization, India

## Abstract

Climate change has intensified heatwaves in India, yet their impact on psychiatric hospitalizations remains underexplored. We conducted a two-year time-series research (2022-2023) at a tertiary care hospital, examining daily admissions for depression and anxiety against meteorological data. Hence, a total of 4,320 psychiatric admissions were recorded, including 1,750 for depression and 2,570 for anxiety. Data shows that heatwave days were associated with modest but significant increases in admissions for both conditions. Women and older adults were more vulnerable. Integrating mental health into heat action plans is warranted.

## Background:

Heatwaves are an increasingly frequent extreme weather event in India and have major health implications [1]. While mortality and cardiovascular risks are well documented, psychiatric consequences have received limited attention [2, 3]. Heat can worsen mental health through disrupted sleep, dehydration, stress hormone imbalance and impaired thermoregulation among patients on psychotropic medications [4, 5]. Studies from high-income countries show increased hospital visits for depression, anxiety and suicide during heat extremes [6, 7-8]. India, with a high baseline prevalence of depression and anxiety [9], is particularly vulnerable. Yet, most Indian studies on heat have examined all-cause hospitalizations or mortality [10, 11], with limited psychiatric focus. Recent policy documents such as the Lancet Countdown report have urged inclusion of mental health outcomes in India's climate adaptation strategies [12]. Therefore, it is of interest to analyze the short-term effect of heatwaves on depression and anxiety hospitalizations at a tertiary hospital over a two-year period.

## Materials and Methods:

This retrospective time-series research was conducted in the psychiatric inpatient unit of a tertiary care hospital in India. Daily hospital admissions for depression (ICD-10 F32-F33) and anxiety disorders (F40-F41) were extracted from hospital records. Meteorological data were obtained from the Indian Meteorological Department. "Definition of heatwave: Per IMD, heatwaves were defined as ≥3 consecutive days with maximum temperature ≥40°C and ≥4.5°C above normal." Daily admission counts were analyzed using Poisson regression with distributed lag non-linear models (DLNM). Adjustments included seasonality, long-term trend, weekday and holidays. Subgroup analyses were stratified by sex and age (<45 vs ≥45 years).

## Results:

During the 2-year period, 4,320 psychiatric admissions occurred, of which 1,750 were for depression and 2,570 for anxiety. A total of 34 heatwave days were recorded. On heatwave days, daily admissions for depression rose from 2.3 to 2.6, a 13% increase (RR 1.13, 95% CI 1.02-1.26). Anxiety admissions increased from 3.4 to 3.7 per day, a 9% rise (RR 1.09, 95% CI 1.01-1.19). Combined psychiatric admissions rose by 11% (RR 1.11, 95% CI 1.04-1.19). These results indicate modest but statistically significant increases in hospitalizations for both depression and anxiety during heatwaves compared to non-heatwave periods (Table 1 - see PDF). Subgroup analysis revealed that women experienced a 14% overall excess risk during heatwaves compared with 8% in men. Adult's ≥45 years were especially vulnerable; with a 17% increase in depression-related admissions and 13% increase in anxiety admissions. Younger adults showed smaller, borderline-significant effects. These findings highlight differential vulnerability across demographic groups, suggesting that both biological and social determinants play a role in amplifying psychiatric risks during heatwaves (Table 2 - see PDF).

## Discussion:

This two-year hospital-based research provides evidence that heatwaves are associated with increased psychiatric hospitalizations for depression and anxiety. The magnitude of risk (RR ~1.09-1.13) is modest but aligns with international literature [6, 7-8]. Our findings emphasize that vulnerable groups, particularly women and older adults, bear disproportionate risk, which is consistent with prior studies showing higher heat sensitivity in these populations [7, 12]. Biological mechanisms include impaired thermoregulation, dehydration and neurochemical stress responses [4, 5]. Psychotropic medication use may further predispose patients to adverse effects [10]. Social factors such as limited access to cooling spaces may amplify vulnerability in elderly and female populations [9]. From a policy perspective, hospital preparedness for heatwaves should extend beyond physical illnesses to include mental health. Awareness programs, medication reviews and integration of psychiatric services into local heat action plans are warranted [12, 13, 14- 15]. Strengths of this research include real-world hospital data, standardized heatwave definition and time-series modeling. Limitations include single-centre design, modest sample size and exclusion of outpatient visits. Future multicentric work could expand the evidence base and assess long-term outcomes.

## Conclusion:

Heatwaves were associated with modest but significant increases in depression and anxiety hospitalizations in a tertiary care hospital in India, with higher risk among women and older adults. Mental health should be explicitly integrated into hospital-level and regional heat action plans.
